# Not just eosinophilic fasciitis

**DOI:** 10.25122/jml-2021-0015

**Published:** 2021

**Authors:** Razvan Chirila, Elena Raluca Cristea, Monica Roxana Purcarea, Laura Carina Tribus

**Affiliations:** 1. Department of Internal Medicine, Mayo Clinic, Jacksonville, Florida, United States of America; 2. Faculty of Medicine, Complutense University of Madrid, Spain; 3. Department of Nephrology, “Carol Davila” Clinical Nephrology Hospital, Bucharest, Romania; 4. Department of Gastroenterology, Bucharest Emergency University Hospital; 5. “Carol Davila” University of Medicine and Pharmacy, Bucharest, Romania

**Keywords:** eosinophilic fasciits, dermatomyositis, eosinophilia-myalgia syndrome, scleroderma, CDC – Center for Disease Control and Prevention, DM – Dermatomyositis, MTX – Methotrexate, EF – Eosinophilic fasciitis, EMS – Eosinophilia-myalgia syndrome, MRI – Magnetic Resonance Imaging

## Abstract

This case report describes a rare case of progressive muscle weakness in a patient treated for eosinophilic fasciitis (EF) for many years before being diagnosed with a second autoimmune disease: dermatomyositis. Our case is a report of a 65-year-old male diagnosed with eosinophilic fasciitis 7 years before being evaluated in our service at Mayo Clinic in Jacksonville, Florida, due to progressive muscle weakness despite the chronic treatment with methotrexate. Contrast-enhanced magnetic resonance imaging of the lower extremity showed enhancement throughout the thigh musculature, which led us to pursue biopsies of the fascia and muscle in order to confirm the diagnosis of EF associated with myopathy. This case illustrates the need to consider the possibility of myopathy in patients diagnosed with EF whenever muscle weakness is more prominent than expected.

## Introduction

Eosinophilic fasciitis (EF) is a disorder that can mimic scleroderma. EF is characterized by symmetrical induration of the skin along with rapid and progressive stiffness of the arms, legs, and trunk [1, 2]. Eosinophilia is typically present in the early phases. In contrast to systemic sclerosis, the involvement of internal organs and Raynaud’s phenomenon are not prominent in EF.

The eosinophilia-myalgia syndrome is a pathology described in patients exposed to L-tryptophan used to treat insomnia. Skin manifestations are similar to those seen in EF [3]. However, myalgia is present and severe. Visceral compromise, such as pneumonitis and neuropathy, may occur, different from EF.

Dermatomyositis (DM) is an idiopathic inflammatory myopathy that leads to proximal muscle weakness along with the presence of muscle inflammation [4, 5]. Multiple distinct cutaneous eruptions are seen in DM. Skin manifestations usually precede the development of muscle weakness [6]. Visceral involvement is not uncommon.

We present a case report of a 65-year-old male diagnosed with EF 7 years prior to his initial evaluation in our facility for a second opinion due to progressive fatigue and muscle weakness. Subsequently, he was diagnosed with EF and DM.

## Case report

A 65-year-old white male presented to our clinic for a second opinion. Seven years earlier, he noticed a skin rash over the anterior part of his neck. He was initially evaluated by a dermatologist, who prescribed topical steroids leading to temporary improvement of the lesion. Unfortunately, the rash returned and was associated with the tightening of the skin over his neck.

Three years later, despite the occasional use of topical steroids, the skin tightness started to spread down his body, affecting his chest, upper and lower extremities. The patient underwent multiple skin biopsies, and the histopathological report was consistent with lymphocytic infiltration, possibly EF. Peripheral eosinophilia was also present.

Based on the biopsy report, the patient was referred to a rheumatologist who diagnosed his disease as EF and first prescribed Plaquenil, and then, due to a lack of improvement, switched to 15 mg of methotrexate (MTX) orally once a week, 1 mg of folic acid orally once a day, and prednisone orally as needed. 

Despite being compliant with his regimen, the skin over his body was getting progressively tighter, and was associated with a lack of energy and joint pain that affected his hands, elbows, knees, and feet. The skin around his neck became so indurated that swallowing solid food was difficult. He developed contraction of the upper and lower extremities associated with severe muscle weakness, requiring a wheelchair for daily activities. 

Three years prior to presentation in our clinic, he had developed a diffuse erythematous rash all over his body requiring hospitalization and was subsequently diagnosed with Stevens-Johnson syndrome and was treated with high doses of steroids.

At the time of our evaluation, the patient denied chest pain, skin rash, shortness of breath, Raynaud’s phenomenon, uveitis, episcleritis, mouth ulcers, or blood in his urine or stools. Physical examination showed a debilitated, frail male who appeared older than his age. Eyes, heart, lung, and abdominal examinations were unremarkable. 

**Figure 1.**
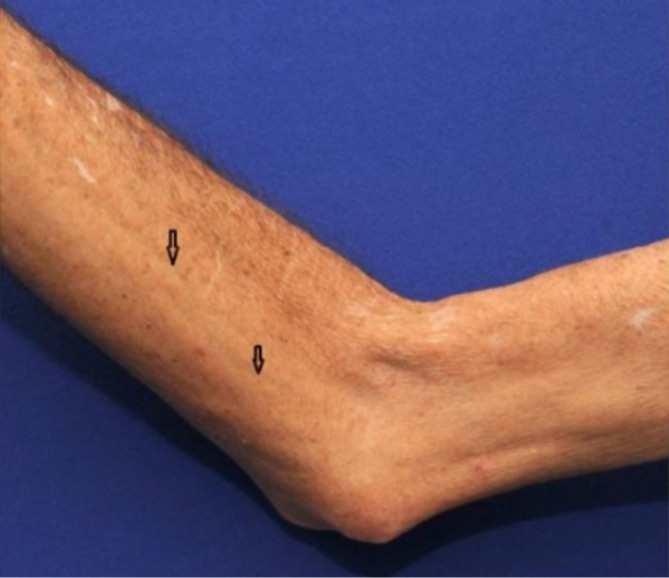
Elbow joint contracture and muscle atrophy. It shows groove signs in the forearm (open arrows).

During the oropharyngeal evaluation, he could not open his mouth fully nor stick his tongue out of the oral cavity. The musculoskeletal evaluation showed diffuse muscle atrophy and proximal and distal muscle weakness (grade 4-/5+) of the upper and lower extremities (Figure 1). Reduced range of motion of the knees, elbows, neck, ankles, and feet associated with severe contraction of these joints was noted. Osteoarthritis of both knees was also noticed. There was no evidence of synovitis.

Skin evaluation demonstrated indurated and thickened skin (peau d’orange) over the face, neck, anterior chest, with the upper and lower extremities extending to the fingers and toes bilaterally. There was a groove sign in both forearms (Figure 1). Neurologically, reduced sensation at the level of both feet was noted Cranial nerves II-XII were intact. Complete blood count, renal function tests, liver enzymes, aldolase, and creatine kinase levels were normal. 

**Figure 2.**
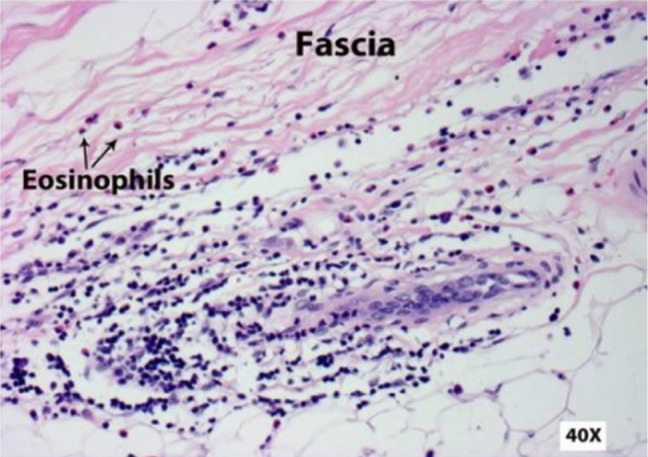
Biopsy of the skin and deep dermis of the thigh, including the adjacent fascia, showed patchy inflammatory infiltrates that included lymphocytes, plasma cells, and eosinophils with a predominance of the inflammation within the fascial layer (40x magnification).

**Figure 3.**
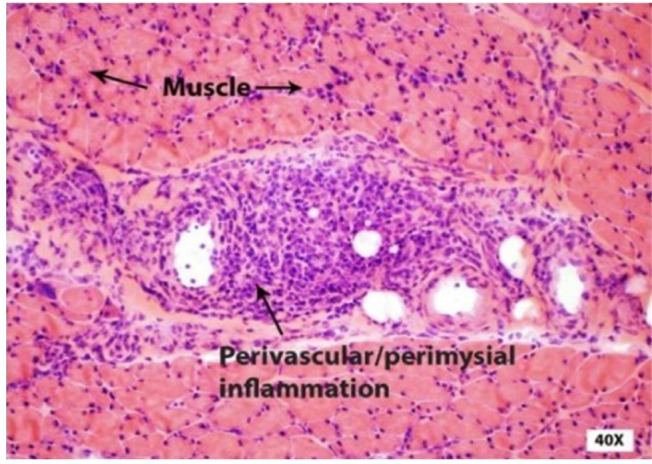
Biopsy of the vastus lateralis muscle demonstrates heavy inflammatory exudate, which is concentrated at perivascular sites in the perimysium (closed arrows) and fascia overlying the muscle. Important perifascicular muscle atrophy is present (40x magnification).

Antinuclear antibodies (ANA), anti-Ro (SSA), anti-La (SSB) antibodies, anti-Smith, anti-Scl-70, anti-centromere, and anti-Jo antibodies were all negative. Erythrocyte sedimentation rate and C-reactive protein were within normal limits. Magnetic resonance imaging (MRI) of the right lower extremity, with and without a contrast agent, showed the presence of diffuse edema and enhancement throughout the thigh musculature. There was no fascial enhancement, abscesses, or evidence of osteomyelitis. Electromyography examination showed the presence of severe sensorimotor polyneuropathic and myopathic processes. Biopsies of the fascia and vastus lateralis muscle are described below (Figures 2 and 3).

Due to the diagnosis of EF and DM, we decided to continue treatment with 15 mg of MTX orally once a week and daily folic acid to control the EF and initiate therapy with two doses of rituximab 1000 mg given 15 days apart in order to treat DM. We also initiated prednisone therapy – 40 mg orally over a month with slow taper due to the severity of muscle weakness.

## Discussion

The presence of skin lesions resembling scleroderma-like disorders, without Raynaud’s phenomenon or internal organ involvement, should raise the possibility of EF [7]. Initially, the main manifestation was non-pitting edema of the skin, followed by induration and development of the peau d’orange appearance [8]. Sclerodactyly is usually absent in EF; however, it was present in our case. It is not unusual for patients to develop joint contractures and neuropathy, and both manifestations were present in our case [7]. Peripheral eosinophilia is a transient manifestation of EF and may only be present in the early course of the disease. Perimyositis may occur due to the involvement of deep skin and fascia. However, inflammatory myositis is uncommon. Creatine kinase levels are typically normal [8].

MRI commonly demonstrate an increased T2 signal in the subcutaneous and deep fascia. Fat-suppressed T1 shows enhancement of the same structures after administration of gadolinium [8].

In clinically suspected EF cases, MRI findings support the diagnosis in the active phase of the disease but should always be confirmed by fascial biopsy [7]. The fascial biopsy and skin examination established the diagnosis of EF in our patient.

The eosinophilia-myalgia syndrome (EMS) is characterized by subacute onset of myalgias and peripheral eosinophilia, followed by chronic neuropathy and skin induration [9]. The skin usually does not involve the fingers and toes. The Center for Disease Control and Prevention (CDC) states that a case definition of EMS is:

1. peripheral eosinophil count of at least 1.0 x 10**9** cells/L;
2. generalized myalgia at some point during the illness that is severe enough to affect the patient’s ability to perform his usual daily activities;
3. no evidence of infection or neoplasia that could explain the eosinophilia or myalgia.

The criteria were not developed with the intent to be applied in clinical diagnosis; they were created for the purpose of surveillance [9]. In our case, the MRI findings and biopsy report were not consistent with EMS.

Our patient had a clinical picture of EF and severe inflammatory myopathy, except for the presence of sclerodactyly seen during the physical exam, which is unusual in EF. The imaging study was compatible only with inflammatory myopathy; however, we believe that the lack of enhancement in the fascia was due to long-term use of MTX since case reports have shown decreased fascial enhancement with MTX therapy [10]. Even with an imaging study consistent with inflammatory myopathy, we decided to proceed with biopsies of two different sites – one from the fascia on the right thigh and the other from the vastus lateralis muscle. The biopsies confirmed the diagnosis of EF and DM. The patient had skin findings similar to scleroderma, but he did not have Raynaud’s phenomenon, and the biopsies were not consistent with the diagnosis of scleroderma.

Rituximab was provided for the treatment of DM, not EF. Our choice was based on our clinical experience and literature studies, which showed that rituximab could be used for DM [11]. It is possible that the rash that developed over three years ago was not Stevens-Johnson syndrome but the initial skin manifestation of DM.

During the last visit, the patient arrived in the room walking without assistance and had gained 15 pounds in weight. The musculoskeletal evaluation showed improvement of muscle atrophy, and the proximal and distal muscle weakness also showed improvement (grade 4+/5+) in the upper and lower extremities. There have been no changes to the skin to date. 

## Conclusion

This report described a rare case of progressive muscle weakness and inflammatory myositis in a patient treated for many years for EF before being diagnosed with dermatomyositis. We woud like to emphasize that myopathy should be considered in a patient diagnosed with EF whenever musculoskeletal symptoms are present.

## Acknowledgment

### Conflict of interest

The authors declare that there is no conflict of interest.
